# Quyu Shengji Formula Facilitates Diabetic Wound Healing via Inhibiting the Expression of Prostaglandin Transporter

**DOI:** 10.1155/2021/8849935

**Published:** 2021-01-22

**Authors:** Yi Lu, Xiaojie Ding, Fei Qi, Yi Ru, Le Kuai, Siting Chen, Yingyao Yang, Xin Li, Fulun Li, Bin Li, Mi Zhou, Kan Ze

**Affiliations:** ^1^Department of Dermatology, Yueyang Hospital of Integrated Traditional Chinese and Western Medicine, Shanghai University of Traditional Chinese Medicine/Institute of Dermatology, Shanghai Academy of Traditional Chinese Medicine, Shanghai 201203, China; ^2^Department of Vascular, Shanghai Hospital of Integrated Traditional Chinese and Western Medicine, Shanghai University of Traditional Chinese Medicine, Shanghai 200082, China

## Abstract

**Background:**

Quyu Shengji Formula (QSF), a Chinese medicine formula widely used in the clinic, has proven therapeutic effects on diabetic ulcers. Nevertheless, the potential mechanism of how QSF cures diabetic ulcer remains elusive.

**Objective:**

To assess the mechanism of QSF against wound healing defects in diabetes.

**Methods:**

Db/db mice were adopted to determine the therapeutic potential of QSF. Further histology analysis was performed by hematoxylin and eosin (H&E) staining. Moreover, the expression patterns of prostaglandin transporter (PGT), prostaglandin E_2_ (PGE_2_), and angiogenesis factor vascular endothelial growth factor (VEGF) were evaluated by immunostaining (IHC) analysis, ELISA assay, real-time quantitative polymerase chain reaction (RT-qPCR), and western blot analysis *in vivo*. Human dermal microvascular endothelial cells (HDMECs) and the shRNA interference technique were used to explore the effects of QSF on cell migration, PGT, PGE_2_, and angiogenesis factor VEGF *in vitro*.

**Results:**

Applied QSF on the wound of db/db mice significantly accelerated wound closure. Reductions of PGT and elevations of PGE_2_ and increased angiogenesis factor VEGF levels were shown after QSF treatment *in vivo* and *in vitro.* Furthermore, QSF promoted HDMEC migration. Inhibition of the expression of PGT by shRNA reversed phenotypes of QSF treatment *in vitro*.

**Conclusion:**

Taken together, our findings reveal that QSF ameliorates diabetes-associated wound healing defects by abolishing the expression of PGT.

## 1. Introduction

A diabetic ulcer is a serious complication of diabetes mellitus. Ulcers are often difficult to heal for a long time and easily lead to amputation and eventual disability. It has been reported that approximately 6.3% of the world's population suffers from diabetes [1]; moreover, a diabetic patient is found with amputation approaching every 30 seconds worldwide [2]. These facts highlight the urgency for scientific discovery-based therapeutic interventions [3].

Debridement and dressing change are often ineffective in treating diabetic ulcers and always cost a lot, which imposes a heavy body-heart burden on the patient, family, and even the whole society. However, some newly developed biological agents and new excipients are expensive or have poor efficacy, and some even pose an oncogenic risk [4]. Traditional Chinese medicine (TCM) has accumulated extensive experience in the repair and diagnosis of wound healing. Traditional experience has created several drugs based on the theory of “Qufu Shengji” (removing slough and promoting the growth of tissue regeneration). We summarized the clinical experience and found that “blood stasis” is also the important pathologic factors of diabetic ulcers. Based on the theory of “Quyu Shengji” (removing blood stasis and promoting the growth of tissue regeneration), we created the “Quyu Shengji” Formula (QSF). QSF has obtained a positive curative effect in the clinic and found that QSF plays a critical role in the regulation of collagen metabolism, MMP-3, and Activin/Follistatin [5–8].

Prostaglandin E_2_ (PGE_2_) has a wide range of physiological functions, especially in the functions of dilating blood vessels and increasing blood flow. Recent studies showed that it has a considerable effect on the healing of gastric ulcers [9]. PGE_2_ level is decreased in chronic diabetic ulcer, which may be an essential reason why diabetic ulcer is difficult to heal [10]. However, the underlying mechanisms remain unclear. PGE_2_ is synthesized by cyclooxygenase (COX) and transported by prostaglandin transporter (PGT) to intracellular degradation. Recent research showed that COX increases in diabetic ulcer wounds [11]. Notably, the reason for the decrease of PGE_2_ can only be analyzed from the perspective of metabolism.

PGT was found relatively late, and its research is still at a relatively preliminary stage. PGT and PGE_2_ showed an obvious negative correlation, and reducing PGT could increase the content of PGE [12], and vice versa. It plays an important role in fever, pregnancy, and acromegaly [13–15]. Studies have revealed that the increased expression of PGT in chronic diabetic ulcers contributes to increased degradation of PGE_2_, leading to a decrease in local PGE_2_ content, which is a crucial reason for delayed wound healing. Inhibition of PGT can facilitate wound healing of diabetic ulcers [12].

Here, we created QSF based on the theory of “Quyu Shengji.” “Quyu” means “removing blood stasis.” In diabetic chronic skin ulcers, the increase in PGT causes a decrease in PGE_2_, leading to local occlusion of blood vessels similar to what TCM calls “Yu” (blood stasis). We adopted db/db mice and human microvascular endothelial cells (HDMECs) as the research object to evaluate QSF regulating the expression of PGT during diabetic healing time and its effects on the metabolism of PGE_2_ and VEGF.

## 2. Materials and Methods

### 2.1. Plant Material

*Drug Preparation*. QSF was comprised of 8 Chinese herbs, as shown in Table 1. The dosage used in the present study was determined according to the Chinese Pharmacopoeia (2015 edition). The herbs were obtained from the pharmacy department of Yueyang Hospital of Integrated Traditional Chinese and Western Medicine, Shanghai University of Traditional Chinese Medicine.

The original medicinal materials were decocted twice in water according to the conventional method of decocting traditional Chinese medicine, then filtered and concentrated into 10 g/ml medicinal solution, added with 70% ethanol for precipitation, and left for 24 hours. After the ethanol is recovered, it is diluted with an appropriate amount of water to make a mixture and finally made into a crude drug-containing 2 g/ml [16]. All the pharmaceutical processes are prepared by the Pharmaceutical Preparation Room of Yueyang Hospital of Integrated Traditional Chinese and Western Medicine Affiliated with Shanghai University of Traditional Chinese Medicine.

### 2.2. Animals

Female clean-grade db/db mice and C57 mice, weighing 20 ± 4 g, were provided by the Experimental Animal Center of the Chinese Academy of Sciences, Shanghai. Animals were maintained under specific pathogen-free conditions. All experiments were performed according to the guidelines of the Committee on Protection, Welfare and Ethics of Experimental Animals in Yueyang Hospital of Integrated Traditional Chinese and Western Medicine affiliated to Shanghai University of Traditional Chinese Medicine (No. YYLAC-2019-043).

### 2.3. Wound Model

All mice were anesthetized by intraperitoneal injection of 0.1% ketamine hydrochloride injection (100 mg/kg). The modeling area was marked with a stamp with a diameter of 8 mm, and both sides of the back spine were shaved with a mechanical razor. To form an open wound with a full-thickness skin defect with a diameter of 8 mm under strict aseptic operation, the left and right sides of the mouse spine were cut to the subcutaneous fascia layer. There are 4 mouse wound models with skin ulcers on the left and right sides [17].

### 2.4. Animal Grouping and Administration Method

After adaptive feeding for 1 week, 12 mice were randomly divided into 3 groups as follows: blank group, control group, and QSF group. The adult dose of QSF was 130 g per day according to the adult standard weight of 70 kg, the mouse coefficient was 9, and the daily dose of rats was 16.7 *μ*g/g. An equal volume of distilled water was administered by gavage in the blank and control groups. The intragastric volume was calculated by 2 g/ml once a day.

We repeated the experiment three times, sacrificing animals on day 1, day 5, and day 9, respectively.

### 2.5. Macroscopic Evaluation

The measurement of the wound area was performed as follows: the quick wound healing adhesive plaster was pressed to the ulceration surface, and a marking pen was used to draw the outline of the wound on the adhesive plaster. A photo of the plaster was taken using a digital camera against a white background. Wound area measurement: then the photo was evaluated by calculating the wound area with image analysis software Image J v1.42q (software is freely downloaded from the National Medical Center website). The wound area on day 1, day 3, day 5, and day 9 after modeling was compared.

### 2.6. H&E and Immunochemistry

On day 1, day 5, and day 9 after the wounding, animals were euthanized by CO_2_ and the tissues of the skin wounds were taken with an 8 mm diameter metallic punch. The tissue was put into liquid nitrogen immediately and kept at −80°C.

HE and immunohistochemical staining were carried out according to the relevant protocols. For general histological analyses, tissue samples were fixed in 10% neutral-buffered formalin, embedded in paraffin, sectioned from the midline of wounds, and stained with H&E.

The expression of PGT in wound tissues was determined with immunohistochemical analysis and the following antibodies: rabbit anti-PGT (diluted: 1 : 150; all from BioTNT, Shanghai, China), and the secondary antibodies goat anti-rabbit IgG HRP (diluted: 1 : 2500). Under the same experimental conditions as the negative control, each sample was diluted and incubated with the same type of antibody. Images were acquired using a light microscope (Olympus, Tokyo, Japan) and analyzed with the image processing software (Image-Pro Plus 6.0).

IHC average optical density value analysis method: each slice in each group was randomly selected with at least 3 200x fields of view for photography. The same brown color was selected as the unified criterion for judging the positivity of all photos by using Image-Pro Plus 6.0. The cumulative optical density value (IOD) and the pixel area (AREA) of each photo were obtained by analyzing each photo. Calculate the average optical density value (AO value); AO = IOD/AREA; the higher the AO value, the higher the positive expression level.

### 2.7. RT-qPCR

The sampling procedure is the same as above. Extracting total RNA: the homogenate was taken and total RNA was extracted according to the Trizol reagent kit method and steps (Invitrogen, Carlsbad, CA, USA). The concentration of total RNA was measured by ultraviolet spectrophotometer. The purity of RNA was measured with agarose gel electrophoresis. Reverse transcribed to synthesize cDNA, primers used were as Table 2.

Reverse Transcription System First Strand cDNA Synthesis Kit was used, with a total reaction volume of 20.0 *μ*l. Real-time PCR amplification: SYBR Green kit (Thermo, Eugene, OR, USA) was used to make real-time PCR amplification of GAPDH, PGT, and VEGF. Real-time fluorescent PCR was used to make a real-time fluorescent quantitative PCR reaction (Applied Biosystems, Foster City, CA, USA). After the reaction was finished, fluorescent quantitative data were collected and analyzed. The data included the amplification curve, working curve, dissociation curve, and corresponding CT value. Light Cycler Software (Version 3.5) was used for data analysis.

### 2.8. ELISA

The levels of PGE_2_ in wound tissue or cell supernatant were determined using ELISA (sensitivity 0.8–300 pg/mL; R&D Systems, Minneapolis, MN, USA; ELISA kits were from BioTNT, Shanghai, China) according to the manufacturer's instructions. Set the blank to zero and measure the absorbance (OD value) of each hole in sequence at 450nm wavelength.

### 2.9. Western Blot

The tissues were lysed and the supernatant was used for protein quantification. GAPDH was used as a protein loading control. The following primary antibodies were used in subsequent experiments: antibody PGT (from Biorbyt, UK) and VEGF antibody (from Abcam, Cambridge, Massachusetts, USA). The secondary antibodies used are goat anti-rabbit HRP and goat anti-mouse HRP (from Biyuntian, Shanghai, China). A Tanon-5200 imaging system (Tanon Science and Technology Co., Shanghai, China) was used to check the target protein and was observed with autoradiography film.

### 2.10. Drug Serum

Male clean-grade SD rats, weighing 250 ± 20 g, were provided by the Experimental Animal Center of the Chinese Academy of Sciences, Shanghai. Animals were maintained under specific pathogen-free conditions. When QSF was given by gavage for 7 days continuously, the rats were killed by CO_2_. Blood samples were taken from the heart aseptically. Samples were left at room temperature for 3-4 hours. They were centrifuged for 10 min at 3000 revolutions/min. Then, we took the supernatant, and it was inactivated in a water bath at 56°C for 30 minutes. We filtered the serum through a 22 *μ*m filter membrane in an ultraclean table to remove bacteria. Serum samples were dispensed into autoclaved centrifuge tubes. The drug serum was stored at −20°C [18].

### 2.11. Cell Culture and Administration Method

Human dermal microvascular endothelial cells (HDMECs) were purchased from Kilton Biotechnology (Shanghai) Co., Ltd. The experiment is grouped as follows:

- Low glucose (blank) group: 5 mmol/L glucose + 10% blank serum
- High glucose (control) group: 25 mmol/L glucose + 10% blank serum
- QSF prescription medicine serum group: 25 mmol/L glucose + 10% QSF drug serum

HDMECs were cultured in endothelial cell medium (ECM) containing 5% fetal bovine serum, 1% endothelial cell growth supplement (ScienCell Research Laboratories), 1% penicillin-streptomycin, and 5 or 25 mmol/L glucose.

Each group had 4 wells; each group was seeded in the corresponding glucose environment at 37°C for 3 days. Medicated serum was added and cultured for 12 hours and then digested with trypsin. Cells were collected for further experiment.

### 2.12. Establishment of the Lentiviral Vector Carrying PGT shRNA

We completed the design of the target gene interference target and then sent it to the gene synthesis company (Kilton Biotechnology Co., Ltd. Shanghai, China) to synthesize the interference target shRNA as Table 3 and completed the shRNA double-strand annealing after synthesis. The vector was linearized and recovered, connected with the annealed shRNA, and transformed by the electrochemical method to complete the identification of positive clones. Recombination interferes with lentivirus packaging according to the requirements of the kit. The transfection experiment was completed, and the cell pellet was collected 48 hours after transfection for PCR experiment to screen the sites with better interference effect. PCR identifies the shRNA with the best interference efficiency and then uses this shRNA for subsequent experiments. Based on the front result, they were divided into a blank group (high glucose, no intervention), shPGT group (high glucose + shPGT interference, interference group), QSF group (high glucose + QSF drug serum), and shPGT + QSF group (high glucose + shPGT interference + QSF drug serum).

### 2.13. Transwell

Briefly, we infiltrate the chamber before inoculation. We put the Transwell chamber into the culture plate, prepared the cell suspension, and inoculated the cells. Cells were fixed at room temperature and observed after staining.

### 2.14. Statistical Analysis

GraphPad Prism 8 (GraphPad Inc., CA, USA) software was used for statistical analysis. The measurement data was expressed by *x* ± SD, and the count data was expressed by the rate. If multiple groups of measurement data conform to the normal distribution (Shapiro-Wilk, SW method) and the variances were uniform, the analysis of variance and Tukey's method for pairwise comparison would be applied. The nonparametric test was used for those who do not meet the conditions of the variance test and Mann–Whitney U method. The Kruskal-Wallis method was applied for the comparison of multiple groups, and the Dunnett method was used for the comparison between groups.

## 3. Results

### 3.1. QSF Promoted Diabetic Wound Healing in db/db Mice

**Figure 1:**
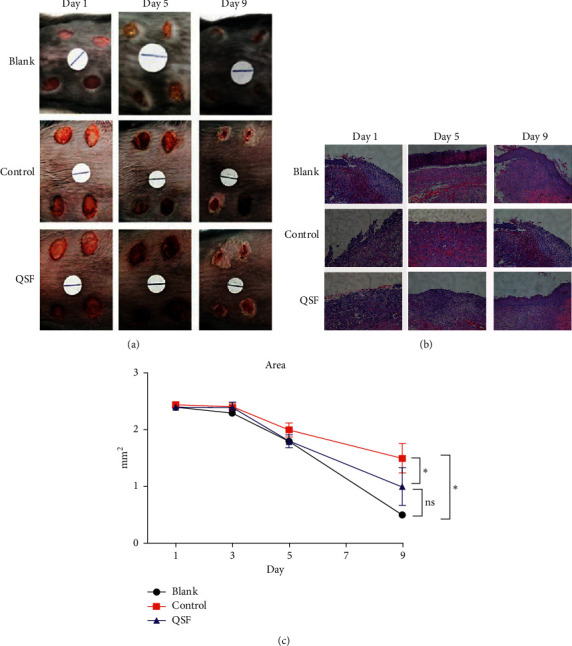
(a) Photographic representation of wound closure on different postwounding days in the blank, control, and QSF groups. Wounding days start from day 1, day 5, and day 9, respectively. (b) Wounded skin by H&E staining in the same groups. Scale = 100 *μ*m. (c) Wound area in the blank, control, and QSF groups from day 1, day 3, day 5, and day 9. ^*∗*^*P* < 0.05, blank vs QSF and control.

To test if QSF can overcome wound healing defects seen in db/db mice, we examined the topical application of QSF *in vivo*. From Figures 1(a) and 1(c), it was obvious that wound healing in the control group composed of db/db mice was more difficult than that that in the blank group with normal blood glucose, while wound healing in the QSF group was better than that in the control group. In particular, wound healing accelerated on day 5 after wounding. Histologically, it was obvious that the infiltration of inflammatory cells in the QSF group was less than that in the control group, while the tissue growth and reepithelialization of QSF were superior to those in the control group (Figure 1(b)). Therefore, from a superficial point of view, we found that QSF could promote wound healing in db/db mice. The effect of QSF on promoting the healing of diabetic wound model was accelerated with the progress of time, which was obvious in the middle and late stages of wound healing.

### 3.2. QSF Inhibited the Expression of PGT and Upregulated PGE_2_ and VEGF in Wound Healing of db/db Mice

**Figure 2:**
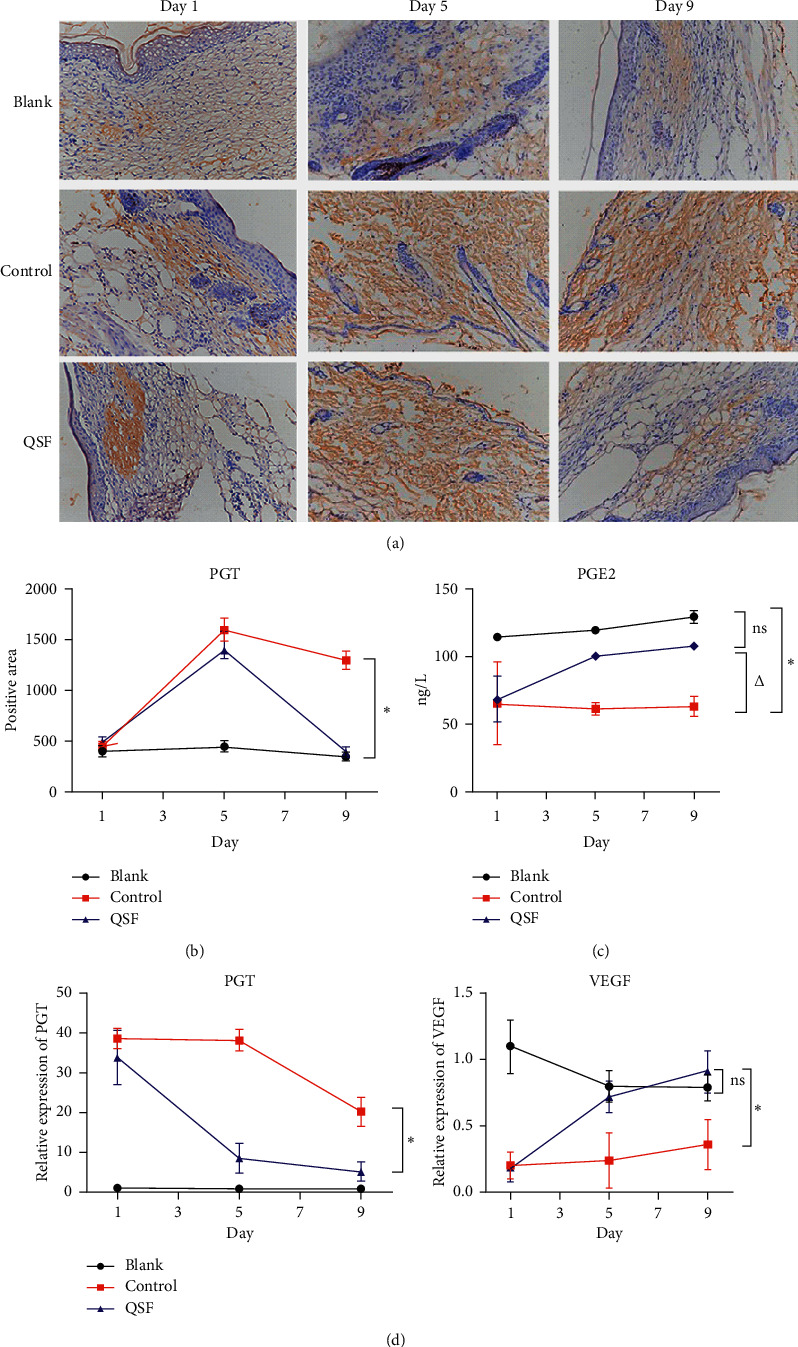
(a) The pictures of immunochemistry of PGT in the blank, control, and QSF groups of day 1, day 5, and day 9. Scale = 100 *μ*m. (b) Positive area of PGT in the same groups of day 1, day 5, and day 9. ^*∗*^*P* < 0.05, control vs QSF and blank. (c) The concentration of PGE_2_ in the same groups of day 1, day 5, and day 9. ^*∗*^*P* < 0.05, control vs QSF. (d) Relative expression of PGT and VEGF in the same groups of day 1, day 5, and day 9. ^*∗*^*P* < 0.05, control vs QSF.

Next, we examined whether the observed accelerated wound closure of QSF wounds could, at least in part, contribute to the inhibition of PGT and the upregulation of PGE_2_ and VEGF. We detected the expression of the molecules mentioned above. As shown in Figure 2(c), compared with the blank group, PGE_2_ content in the control group was significantly decreased, while that in the QSF group increased PGE_2_ content. Such an effect even appeared in the middle stage of wound healing.

**Figure 3:**
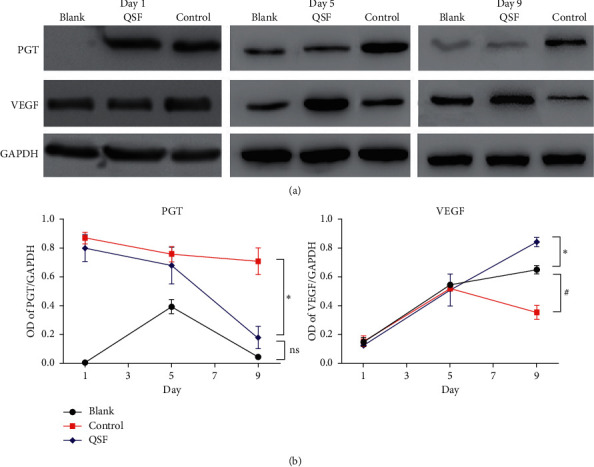
(a) Protein level of PGT and VEGF by western blot in the blank, control, and QSF groups of day1, day 5, and day 9. (b) OD of PGT/GAPDH and VEGF/GAPDH in the same groups. ^*∗*^*P* < 0.05, control vs. QSF. #*P* < 0.05 control vs blank.

From Figures 2(a), 2(b), 2(d), and 3, IHC, PCR, and WB were used to verify that in the db/db mouse ulcer model, tissue PGT expression increased significantly, while VEGF expression decreased. The QSF group inhibited the high expression of PGT and increased the expression of VEGF, and this effect began to occur as early as the middle stage of wound healing.

### 3.3. QSF Improved the Migration Ability of HDMEC and Regulated PGE_2_, PGT, and VEGF

**Figure 4:**
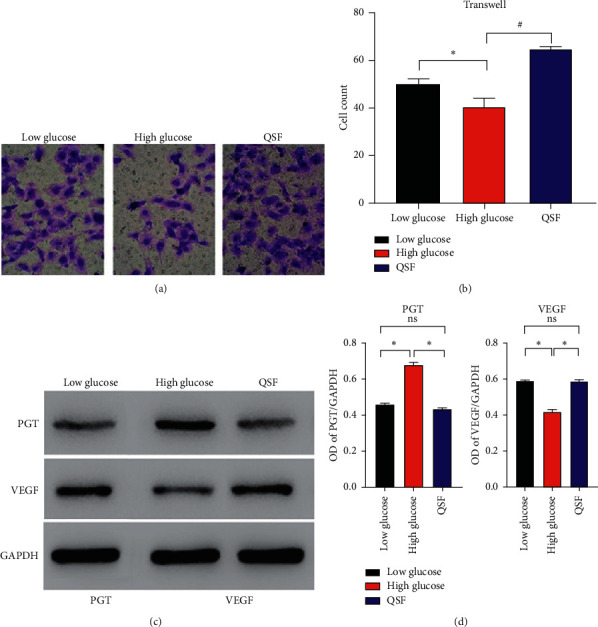
(a) The pictures of Transwell in the low glucose, high glucose, and QSF groups. (b) Cell count in the same groups, ^*∗*^*P* < 0.05, high glucose vs QSF and low glucose. (c) The protein level of PGT and VEGF in the same groups. (d) OD of PGT/GAPDH and VEGF/GAPDH in the same groups. ^*∗*^*P* < 0.05, high glucose vs QSF and low glucose.

Due to HDMEC's ability to improve local angiogenesis of the wound and enhancing blood supply, we examined whether QSF affects the migration ability of HDMEC. Through Figures 4(a) and 4(b), we found that the migration capacity of HDMECs under high-glucose environment feeding was reduced, and this result could be corrected by QSF. Meanwhile, the protein expression of PGT in the high-glucose environment was significantly higher than that in the low-glucose environment, while the expression of VEGF was significantly decreased. QSF reduced the PGT expression of HDMEC and improved VEGF in the high-glucose environment (Figures 4(c) and 4(d)).

### 3.4. The Ability of QSF on Cell Migration and Regulation PGE_2_, PGT, and VEGF Was Decreased after PGT Was Blocked

**Figure 5:**
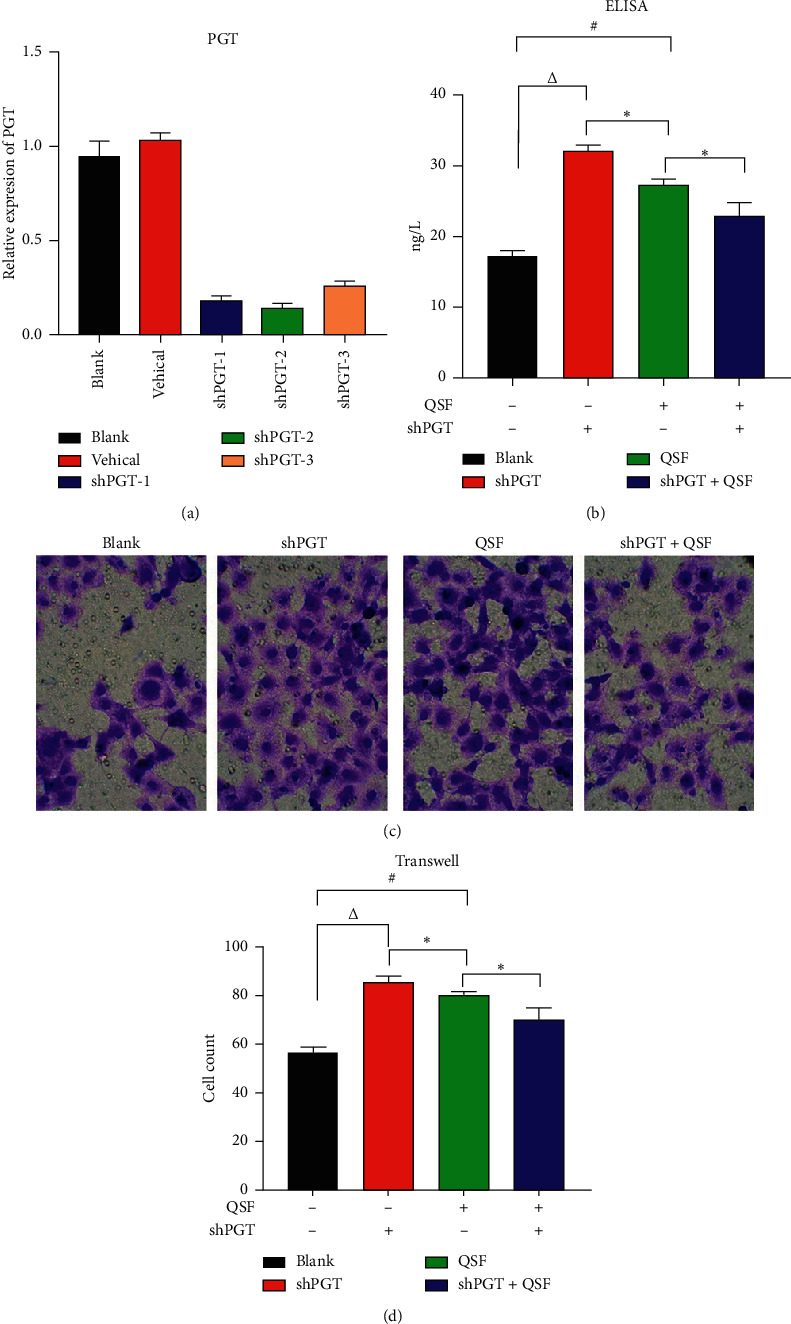
(a) Relative expression of PGT in the blank, vehicle, shRNA-PGT-1, shRNA-PGT-2, and shRNA-PGT-3 groups. (b) The concentration of PGE_2_ in the blank, shRNA-PGT, QSF, and shRNA-PGT combined with QSF groups. ^*∗*^*P* < 0.05, QSF group vs shRNA + QSF and shRNA group; #*P* < 0.05, QSF vs blank; Δ*P* < 0.05, shPGT group vs blank group. (c) The pictures of Transwell in the same groups. (d) Cell count in the same groups. ^*∗*^*P* < 0.05, QSF group vs shRNA + QSF group; #*P* < 0.05, QSF vs blank; Δ*P* < 0.05, shPGT group vs blank group.

Three shRNA sequences were used for the validation test. It was found in Figure 5(a) that empty vector had no effect on PGT expression, while shPGT-2 had the best inhibitory efficiency, so we chose shPGT-2 for subsequent study.

By the ELISA test, we found that after shPGT inhibited the expression of PGT, the content of PGE_2_ increased significantly, and the QSF group also achieved similar results. However, if shPGT inhibited the expression of PGT, QSF did not play a role as before, indicating that the effectiveness of QSF depended on PGT to a certain extent. Interestingly, similar results were obtained in the Transwell test in the same situation, which also indicates that the effect QSF group is to some extent dependent on the expression of PGT.

## 4. Discussion

Prostaglandin transporter (PGT) is the main protein that mediates the transmembrane transport of PGs such as PGE_2_. It is difficult for PGs to pass through the cell membrane in a freely diffused manner [14, 19]. Therefore, PGT mediates the transmembrane transport of PG through the lactate-PG transport mechanism and is the main transport carrier of PG. PGT can transport PGs outside the cell membrane into the cell membrane, thereby inactivating PGs outside the cell membrane [20]. Previous studies have found that for the extracellular environment, increasing the glucose concentration will reduce PGE_2_, while decreasing the glucose concentration will increase PGE_2_ [21].

In recent years, studies on diabetic ulcer wounds have shown that increased tissue glucose content will cause PGT-mediated reduction of PGE_2_ content, leading to a series of pathological changes such as local tissue capillary occlusion and local tissue hypoxia [22, 23].

PGE_2_ is transferred into the cell through the intercellular PG transporter and is rapidly inactivated by 15-ketoprostaglandin (15-PGDH). In diabetic animals, PGE_2_ synthase did not decrease, but the expression of PGE2 decreased, which made the wound difficult to heal. High glucose levels will increase PGT and accelerate PGE_2_ metabolism. PGT inhibitors can accelerate wound healing and increase VEGF expression [24, 25].

The project that the author participated in the early stage partly clarified the mechanism of TCM on wound healing from the perspectives of the “stasis-removing and tissue-generating” method regulating growth factors, collagen metabolism, MAPK, and Wnt/*β*-catenin signaling pathways [5–8, 17]. In the process of clinical diagnosis and treatment, the improvement of QSF on wound healing of diabetic patients has been fully affirmed by patients, but compared with the complex human environment, there are still many shortcomings in cell-level experiments. TCM also improves wound healing in multiple ways and targets. To further improve the theory of removing blood stasis and promoting muscle growth, more detailed cell pathway exploration is needed.

We created QSF based on the theory of “Quyu Shengji.” “Quyu” means “removing blood stasis.” In diabetic chronic skin ulcers, the increase in PGT causes a decrease in PGE2, leading to local occlusion of blood vessels similar to what TCM calls “Yu” (blood stasis). The theory of “Quyu” (removing blood stasis) is one of the main basic theories of TCM. In particular, great progress has been made in the past 200 years, making certain new treatment methods available for diseases that were difficult to treat in the past [26–29].

In the current study, db/db mouse skin ulcer model was used as the research object. It was found that, compared with the blank group, the control group had difficulty in wound healing, impaired local blood supply, decreased tissue PGE_2_ and VEGF expression, and increased PGT expression. The traditional Chinese medicine QSF Decoction improves the series of pathological changes by inhibiting the expression of PGT and increasing PGE_2_ and VEGF *in vivo* and *in vitro*.

## 5. Conclusion

In summary, QSF attenuated PGT expression which promoted diabetic wound healing. Further studies on the effects of PGT knockout might reveal more molecular roles for PGT in diabetic wound healing. Our future direction is to further investigate the effect of PGT on nonhealing wounds.

## Figures and Tables

**Table 1:** Ingredients of QSF with English translations.

Main composition	Latin scientific name	Plant part (s)	Amount (g)
radix astragali	*Astragalus membranaceus* (Fisch.) Bge. var. mongholicus (Bge.) Hsiao	Root	30
Radix pseudostellariae	*Pseudostellaria heterophylla* (Miq.) Pax et Pax et Hoffm.	Root	9
Rhizoma atractylodis macrocephalae	*Atractylodes macrocephala* Koidz	Root	9
Radix Rehmannia	*Rehmannia glutinosa* Libosch.	Root	12
Radix salviae miltiorrhizae	*Salvia miltiorrhiza* Bge.	Root	30
Hirudo	*Whitmania pigra* Whitman	All	6
Semen Persicae	Prunus persica (L.) Batsch	Seed	9
Rhizoma chuanxiong	Ligusticum chuanxiong Hort.	Root	9

**Table 2:** Primers.

Species	Target	Direction	Sequence
Mouse	PGT	Forward	5-TCGATAGTGGTGAGGCTGCT-3
		Reverse	5-CGCTCGGTCTTCAACAACAT-3

Mouse	VEGF	Forward	5-AATGCTTTCTCCGCTCTGAA-3
		Reverse	5-GCTTCCTACAGCACAGCAGA-3

Mouse	GAPDH	Forward	5-GGGCATCTTGGGCTACACTG-3
		Reverse	5-CATGAGGTCCACCACCCTGT-3

**Table 3:** shRNA design sequence.

PGT locus 1 (370–388)	GGTGTTTGTGCTCTGCCAA

PGT-F	CCGGTGGTGTTTGTGCTCTGCCAACTCGAGTTGGCAGAGCACAAACACCTTTTTG

PGT-R	AATTCAAAAAGGTGTTTGTGCTCTGCCAACTCGAGTTGGCAGAGCACAAACACCA

PGT locus 2 (444–462)	CCACCATTGAGAAGCGCTT

PGT-F	CCGGTCCACCATTGAGAAGCGCTTCTCGAGAAGCGCTTCTCAATGGTGGTTTTTG

PGT-R	AATTCAAAAACCACCATTGAGAAGCGCTTCTCGAGAAGCGCTTCTCAATGGTGGA

PGT locus 3 (1998–2016)	CCATTGACCACTCCTGCAT

PGT-F	CCGGTCCATTGACCACTCCTGCATCTCGAGATGCAGGAGTGGTCAATGGTTTTTG

PGT-R	AATTCAAAAACCATTGACCACTCCTGCATCTCGAGATGCAGGAGTGGTCAATGGA

## Data Availability

The data used to support the findings of this study were supplied by Dr. Kan Ze under license and so cannot be made freely available. Requests for access to these data should be made to Dr. Kan Ze, zekan1@163.com.
